# An Integrated Model of Heat Transfer in Meat Products during Multistage Operations

**DOI:** 10.3390/foods12183369

**Published:** 2023-09-08

**Authors:** Aberham Hailu Feyissa, Stina Frosch

**Affiliations:** Food Production Engineering, National Food Institute, Technical University of Denmark (DTU), 2800 Lyngby, Denmark; froschstina@gmail.com

**Keywords:** cooling process, FEM, heating process, food processing, salted loin pork, smoking process

## Abstract

This work focuses on the modelling of the heat transfer in the key processes during the manufacturing of salted–smoked loin pork, a traditional Danish product called “Hamburgerryg”. Drying, smoking, steam-cooking, water-cooling, and air-cooling processes are important process steps in the production of “Hamburgerryg”. A mathematical model that describes the heat transfer during these processes was developed. A current model formulation, multiple unit operations, and the transfer between these unit operations were considered and described by an equation that combines boundary conditions. The model governing and boundary equations were solved using the finite element method (COMSOL Multi-physics^®^ version 5.6). The product temperature profile during the processes was predicted as a function of position and time in the loin. The model was validated using measured temperature profiles from industrial production, and a good agreement between the measured and simulated temperature profiles was obtained. Additionally, the effects of the position (in the heating, cooking, and cooling chamber) on the temperature profile were also investigated. The obtained model can be used as a simulation tool to predict the temperature profile (particularly cold and hot spots) for entire processes and this can aid in the digitization of food processes by providing a more accurate and efficient means of temperature control.

## 1. Introduction

Thermal processing, heating, and cooling are the key processes in the manufacturing of salted–smoked pork loin, a traditional Danish meat product called “Hamburgerryg”. During the manufacturing of the salted–smoked pork loin, the product undergoes drying, smoking, cooking, and cooling processes. The product temperature profile during these processes is an important variable that determines the final product quality and microbial safety. In particular when processing a big piece of meat such as salted–smoked pork loin, efficient control of the process conditions (e.g., heating, cooking, and cooling processes) and obtaining knowledge of the temperature distribution within the product is a relevant issue for the food industry, which directly affects the product’s safety and quality. Therefore, monitoring and controlling the temperature is commonly accepted as important during the processing of meat products, for example, high cooking temperatures can reduce cooking time, but can also cause greater cooking loss and lower texture quality [1].

The development of a simulation tool for predicting temperature profiles throughout various stages of food processing represents a crucial step toward the digitization of food processes. By implementing digital twins of food processing systems, the model can be used to optimize process efficiency, reduce energy consumption, and minimize waste, all while ensuring the safety and quality of the final product. Modelling of heat transfer during the manufacturing of meat products plays an important role in the prediction of the temperature distribution as a function of time and position and helps to control the time–temperature profile of the product in order to control the quality and safety of the final product. In the literature, there are few publications on the modelling of these processes [2,3,4,5,6] and the related processes of cooling [7,8,9], roasting, and baking in a convection oven [10,11,12,13,14,15,16,17,18,19]. Bottani and Volpi (2009) developed an analytical model for cooking in industrial steam ovens to study time–temperature profiles of meat samples with different sizes [3]. Hu and Sun (2000) studied the cooling rate and weight loss of cooked ham during an air-blast chilling process using computational fluid dynamics (CFD) [4]. Their study only focused on heat and mass transfer during the chilling process. Amézquita et al. (2005) developed an integrated model for heat transfer and microbial growth of Clostridium perfringens during the cooling of cooked boneless ham [2]. Llave et al. (2015) utilized a mass transfer model for the migration of smoke components into pork loin ham during processing and storage [5]. Their study did not include heat transfer. Most of these studies modeled an individual process, not all processing steps, either for the cooling process or for the heating process. None of the studies considered what happens during the transferring time between the process steps (e.g., from the smoking–drying chamber to the cooking chamber, from the cooking chamber to the cooking room). Obtaining a picture of the internal product temperature distribution between the process steps requires models that describe the entire process of operations (e.g., for the production of salted–smoked pork loin this covers drying, smoking, heating, cooling, and transfer between these unit operations). Such models are important to predict the temperature profile as a function of time and position for the whole process, however, these kinds of models are lacking. Modelling of food operations is a key enabler of the use of digitalization (particularly digital twins) in food industry optimization production processes. Therefore, in this paper, we have chosen to develop a single holistic model as the framework to represent available knowledge on heat transfer during the manufacturing of meat products.

The main aim of this work is to make a heat transfer model covering all the processes for the production of salted–smoked loin pork, including transfer from one unit to another, and then to predict the corresponding temperature profile as a function of time and position within the product. In this study, we consider the heat transfer during drying–smoking, steam-cooking, water-cooling, and air-cooling. In addition, we also consider the heat transfer during the transfer time of the product from the drying–smoking chamber to the cooking chamber and from the cooking chamber to the cooling room. During the transfer, the product is exposed to ambient conditions—an ambient air temperature of 25 °C. This means that the surface of the product cools down or heats up depending on the state of the product and the ambient conditions. For example, the surface of the product slightly cools down when the product is transferred from the drying–smoking chamber to the cooking chamber, as the product surface temperature is higher than the ambient temperature. Therefore, it is necessary to include all the process steps (including the transfer time) in the modelling to be able to predict the entire product temperature profile as a function of time and position within the product as each processing step influences the next processing steps in the production.

## 2. Modelling of Heat Transfer during the Processing of Smoked–Salted Loin Pork

### 2.1. Process Description and Model Formulation

**Figure 1:**
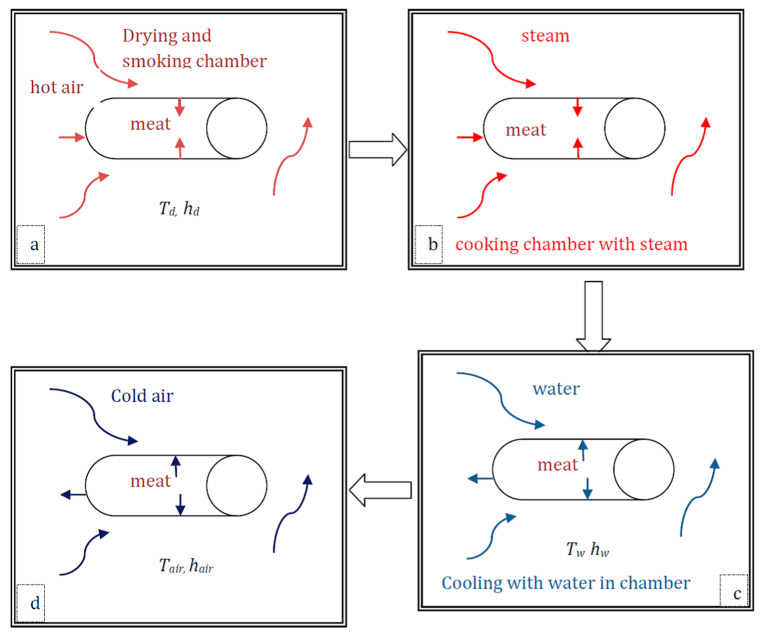
Heat transfer process during the salted–smoked loin pork processing: (**a**) drying–smoking (DS) processes in the smoking chamber, (**b**) steam-cooking (SC) in the cooking chamber, (**c**) water-cooling (WC) in the cooking chamber, and (**d**) air-cooling (AC) in the cooling room. Note: here, only one product is indicated (for better clarity) to illustrate the mechanism of heat transfer (the products and placements are detailed in Figure 2).

The salted loin pork (packed into a fibre casing) is dried and subsequently smoked in the drying–smoking chamber before cooking with steam (Figure 1). The product is then moved into the cooking chamber and heated using steam at 78 °C (351.15 K) for 2.2 h (7920 s). In the same cooking chamber, the product is cooled by spraying water on the product for 2 h (7200 s). The processes and the product interaction during the processing of salted–smoked loin pork are described in Figure 1. There are six stages (four processing steps and two transferring steps) that involve heating and cooling operations.

The first stage is the drying–smoking (DS) process, which includes drying and smoking of the salted–smoked pork loin in the same chamber. During the drying–smoking, the salted pork loin is heated and smoked in the drying–smoking chamber (Figure 1a). The main heat transfer mechanism is the convective heating of the meat product by the circulating hot air. The second stage is transferring from the drying–smoking chamber to the steam-cooking chamber. The third stage is the steam-cooking (SC) of the product. During the steam-cooking, the product is heated in the cooking chamber (Figure 1b), which means that the main heat transfer mechanism is convective heating of the meat product by the circulating steam. The fourth stage is cooling with water (water-cooling). During the water-cooling (WC), the product is cooled in the cooking chamber with water. The main heat transfer mechanism is the convective heating of the meat product by the spraying of water on the product (Figure 1c). The fifth stage is transferring the product from the steam-cooking chamber to the cooling room. The sixth stage is air-cooling (AC), which is cooling with cold air by transferring the product from the cooking chamber to the cool room. During the air-cooling process, the product is chilled in the cool room by the circulating cold air. The main heat transfer mechanism is convective cooling of the product by the circulating cold air (Figure 1d). This study only takes into account heat transfer and not mass transfer, as we assume that the casing used in the research has low permeability and, thus, any mass loss is negligible. This is supported by the fact that water loss in all samples during the smoking process was minimal (less than 4%), as calculated from the weights measured before and after the process (not presented here). It is also worth noting that the chamber used in the study maintained a moderate humidity of 45–50% and was not filled with dry air.

### 2.2. Heat Transfer Equations

#### 2.2.1. Governing Equation

The governing heat transfer equation within the salted–smoked pork loin is assumed to be given by Equation (1) [20]

(1) $$\rho_{m}c_{pm}\frac{\partial T}{\partial t}=\nabla (k_{m}\nabla T)$$

where *ρ_m_* is the density of meat (kg/m^3^), *k_m_* is the thermal conductivity of the meat (W/(m·K)), *c_pm_* is the heat capacity of meat (J/(kg·K)), *T* is the temperature (K), and *t* is time (s).

The heat flux boundary condition is convective heat flux from the heating/cooling to the product surface [16]. The following boundary conditions apply for the heat transfer at different stages of processing.

#### 2.2.2. Boundary Condition

The boundary condition during the drying–smoking process (the first stage) is given by Equation (2):

(2a) $$-n\cdot \left(k_{m}\nabla T\right)=h_{d}\left(T_{d}-T_{s}\right)$$

(2b) $$-n\cdot \left(k_{m}\nabla T\right)=h_{d}\left(T_{smoke}-T_{s}\right)$$

where *h_d_* is the heat transfer coefficient (W/(m^2^·K)) during the drying–smoking and *T_d_* and *T_smoke_* are the process temperature (K) during drying and smoking, respectively.

The boundary condition during the steam-cooking process (third stage) is given by Equation (3):

(3) $$-n\cdot \left(k_{m}\nabla T\right)=h_{st}\left(T_{st}-T_{s}\right)$$

where *h_st_* is the heat transfer coefficient (W/(m^2^·K)) during SC and *T_st_* is the steam temperature (K) during the SC process.

The boundary condition during the water-cooling process (fourth stage) is given by Equation (4):

(4) $$-n\cdot \left(k_{m}\nabla T\right)=h_{w}\left(T_{w}-T_{s}\right)$$

where *h_w_* is the heat transfer coefficient (W/(m^2^·K)) during the water-cooling process and *T_w_* is the water temperature (K) during the water-cooling (process temperature).

The boundary condition during the air-cooling process (sixth stage) is given by Equation (5):

(5) $$-n\cdot \left(k_{m}\nabla T\right)=h_{air}\left(T_{air}-T_{s}\right)$$

where *h_air_* is the heat transfer coefficient (W/(m^2^·K)) during air-cooling and *T_air_* is the air temperature (K) during the AC process.

The boundary condition during the transfer of the product between equipment (which includes the transfer from the DS chamber to the SC chamber (second stage) and from the SC chamber to the AC chamber (fifth stage) is given by Equation (6):

(6) $$-n\cdot \left(k_{m}\nabla T\right)=h_{amb}\left(T_{amb}-T_{s}\right)$$

where *h_amb_* is the heat transfer coefficient (W/(m^2^·K)) during transfer and *T_amb_* is the surrounding/environment air temperature (K) during the transfer time.

In each of the stages, the same type of boundary condition is taken into account (convection boundary condition, Equations (2)–(6)). Thus, all the boundary equations (Equations (2)–(6)) can be combined and written as Equation (7)

(7) $$-n\cdot \left(k_{m}\nabla T\right)=h\left(T_{p}-T_{s}\right)$$

where *h* is the heat transfer coefficient (W/(m^2^·K)) and function of process conditions and *T_p_* is the process temperature (K) and function of process conditions. The surface boundary condition varies with time at different stages of processing as the product is exposed to different process conditions, which are given by Equations (2)–(6). Therefore, in Equation (7), *h* is a function of time and *T_p_* is a function of the time (*t*) at different stages of processing during the production. The h and *T_p_* are described by Equation (8a,b), respectively, as follows:

(8a) $$\begin{array}{l} h=\left\{ \begin{array}{ll} h_{d}=h_{smoke} & \mathrm{if}\;t\leq t_{d}\;\mathrm{and}\;t_{d}<t\leq t_{smoke}+t_{d}\\ h_{amb} & \mathrm{if}\;t_{smoke}+t_{d}<t\leq t_{smoke}+t_{d}+t_{trans1}\\ h_{st} & \mathrm{if}\;t_{smoke}+t_{d}+t_{trans1}<t\leq t_{smoke}+t_{d}+t_{trans1}+t_{st}\\ h_{w} & \mathrm{if}\;t_{smoke}+t_{d}+t_{trans1}+t_{st}<t\leq t_{smoke}+t_{d}+t_{trans1}+t_{st}+t_{w}\\ h_{amb} & \mathrm{if}\;t_{smoke}+t_{d}+t_{trans1}+t_{st}+t_{w}<t\leq t_{smoke}+t_{d}+t_{trans1}+t_{st}+t_{w}+t_{trans2}\\ h_{air} & \mathrm{if}\;t_{smoke}+t_{d}+t_{trans1}+t_{st}+t_{w}+t_{trans2}<t\leq t_{smoke}+t_{d}+t_{trans1}+t_{st}+t_{w}+t_{trans2}+t_{air} \end{array}\right. \end{array}$$

(8b) $$\;T_{p}=\left\{ \begin{array}{ll} T_{d} & \mathrm{if}\;t\leq t_{d}\;\\ T_{smoke} & \mathrm{if}\;t_{d}<t\leq t_{smoke}+t_{d}\\ T_{amb} & \mathrm{if}\;t_{smoke}+t_{d}<t\leq t_{smoke}+t_{d}+t_{trans1}\\ T_{st} & \mathrm{if}\;t_{smoke}+t_{d}+t_{trans1}<t\leq t_{smoke}+t_{d}+t_{trans1}+t_{st}\\ T_{w} & \mathrm{if}\;t_{smoke}+t_{d}+t_{trans1}+t_{st}<t\leq t_{smoke}+t_{d}+t_{trans1}+t_{st}+t_{w}\\ T_{amb} & \mathrm{if}\;t_{smoke}+t_{d}+t_{trans1}+t_{st}+t_{w}<t\leq t_{smoke}+t_{d}+t_{trans1}+t_{st}+t_{w}+t_{trans2}\\ T_{air} & \mathrm{if}\;t_{smoke}+t_{d}+t_{trans1}+t_{st}+t_{w}+t_{trans2}<t\leq t_{smoke}+t_{d}+t_{trans1}+t_{st}+t_{w}+t_{trans2}+t_{air} \end{array}\right.$$

where *t* is the time (s) and *T* is the temperature (K). The values and the description of each parameter (time and temperature) are given in Table 1.

#### 2.2.3. Initial Condition and Thermo-Physical Properties

The initial value of temperature was obtained from the measurements. The initial temperature (To), thermo-physical properties are functions of composition and were determined or estimated from the compositions using the equations provided in Table 1 and all the input parameters are given in Table 1.

### 2.3. Model Solution

**Figure 2:**
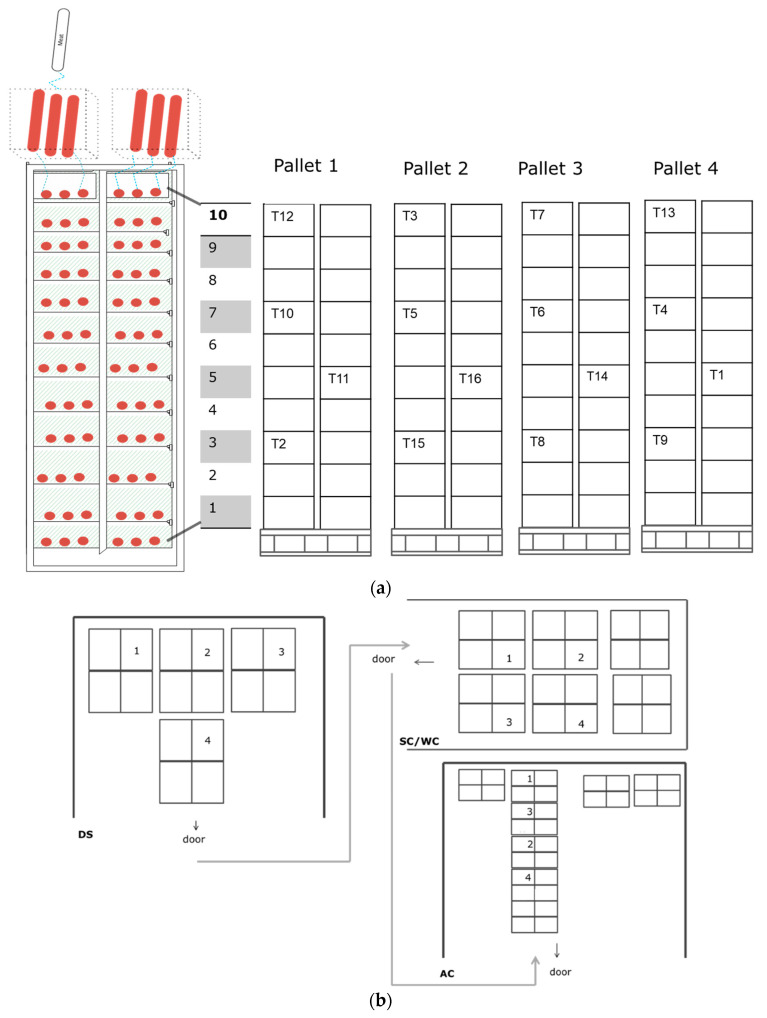
(**a**) Illustrates the placement of meat products (red cylindrical or circles) on the pallet, the position of the data loggers in the packed products at different positions (T12, T10, T11, and T2 in pallet 1; T3, T5, T16, and T15 inpallet 2; T7, T6, T14, and 8 in pallet 3; T13, T4, T1, and T9 inpallet 4), and (**b**) the pallets are placed at different location (1, 2, 3 and 4) within the processing environment. DS: drying–smoking chamber, SC/WC: steam-cooking/water-cooling chamber and AC: air-cooling room. Door is indicating the door of each chamber. Layer numbers are indicated with numbers from bottom to top with 1 to 10 in (**a**).

The mathematical model (Section 2.2) describing the heat transfer in the product during the six stages (two transferring stages and four processes: drying–smoking, steam-cooking, water-cooling, and air-cooling) was implemented and solved using the finite element method (FEM) with COMSOL Multiphyics^®^ version 5.6. Axial symmetry 2D cylindrical geometry of dimensions (length = 270 mm and radius = 42.5 mm)—corresponding to half of the original dimensions (length = 540 mm and diameter = 85 mm)—of the sample was built in COMSOL for numerical simulation. The 2D geometry was chosen on the basis of axial symmetry to simulate the 3D geometry. As a consequence, the computational burden during the simulations was significantly reduced. The geometry was meshed (consisting of 1010 elements, 564 vertices, and average mesh quality of 0.944) and the mesh quality was checked using a mesh sensitivity analysis [26]. A series of simulations were performed with increasingly finer mesh until convergence, i.e., until the change in mesh density no longer had an impact on the solution. The generated mesh was further refined (e.g., at the boundaries where there is a high gradient) to improve the accuracy of the numerical results. The governing partial differential equation of heat transfer together with the boundary equations was solved, and the temperature distribution was predicted as a function of position and time in the product. The temperature measurement at position (T11 in Figure 2) pallet 1 was utilized to estimate the unknown parameter (*h_air_* and *h_w_* heat transfer coefficients), while the remaining temperature measurements were employed for model validation (Section 3.2. The parameters were estimated using the least squares method, which involved comparing the simulated and experimental temperature profiles. The objective function, which was the sum of squared differences between the simulated and measured temperature profiles, was minimized to obtain the resulting parameters. Temperature values were sampled every minute (60 s). For a more detailed account of the method, please consult [14]. The obtained experimental temperature profiles (all positions and pallets) were utilized to compare the model’s prediction for entire process operations. Moreover, to assess the accuracy of the model, statistical measures such as the coefficient of determination (R-squared) and root mean squared error (RMSE) were utilized to compare the model’s predictions with experimental or observational data. In all cases, the R-squared value was found to exceed 0.938, with most cases showing values greater than 0.98. The maximum observed RMSE value was 5.94, but in the majority of cases, it was lower than 2.67 (for detail, see Table 2). The plot of the absolute residual, which represents the difference between the predicted and measured values, also was examined as a function of time (see Section 3.2).

### 2.4. Experimental Data and Model Validation

The salted loin pork meat with a diameter of 85 mm and length of 540 mm was placed in boxes (three pieces in each box) and stacked on pallets as illustrated in Figure 2. Sixteen temperature loggers were placed in different pallets and locations (Figure 2a). The temperature profiles were measured using data loggers (SmartButton datalogger, ACR systems Inc., Surrey, British Columbia, Canada) at the centre of the meat products (salted pork loin) and the salted pork loins were placed at different positions. To be specific, on pallet 1: “T12”, “T10”, “T11”, and “T2”; pallet 2: “T3”, “T5”, “T16”, and “T15”; pallet 3: “T7”, “T6”, “T14”, and “T8”; and pallet 4: “T13”, “T4”, “T1”, and “T9”. This arrangement is depicted in Figure 2. The temperature was measured as function of time in 1 min intervals during the entirety of processing (four main processing steps: drying–smoking, steam-cooking, water-cooling, and air-cooling, and two transferring stages). The measured data were used for validating the proposed heat transfer model (Section 3.2).

## 3. Results

**Figure 3:**
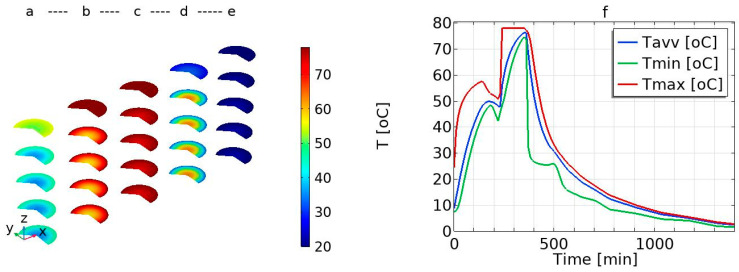
Simulated temperature distribution in the loin pork: (**a**) during drying–cooking at *t* = 100 min, (**b**) during steam-cooking at *t* = 266.67 min, (**c**) during steam-cooking at *t* = 333.33 min, (**d**) the temperature water-cooling at *t* = 400 min, and (**e**) during air-cooling at *t* = 600 min; and (**f**) average volume (*Tavv*), maximum (*Tmax*), and minimum (*Tmin*) simulated loin pork temperature profile.

Figure 3 presents the predicted temperature distribution within the meat product at different time of processing (*t* = 100 min, *t* = 266.67 min, *t* = 333.33 min, *t* = 400 min, and *t* = 600 min). Figure 3a shows the predicted temperature distribution within the meat product at *t* = 100 min during the drying–smoking processes. Figure 3b,c show the predicted temperature distribution within the meat product at two different times during the steam-cooking (*t* = 266.67 min and *t* = 333.33 min), illustrating the progress of the temperature profile during steam-cooking. At *t* = 266.67 min, the surface temperature of the meat product is at a higher temperature compared to the inside part/central region (Figure 3b), but at *t* = 333.33 min, the product temperature distribution is more uniform (Figure 3c). The center temperature or coldest spot temperature at *t =* 333.33 min is 72.4 °C (which is approximately at the minimum criteria for the food safety point = 72 °C). Figure 3d,e illustrate the temperature distribution in the meat product during the water-cooling process (*t =* 400 min) and air-cooling process (*t =* 600 min), respectively. From the illustrations, it is clear that the product is cooled from the outside and, as expected, the temperature gradient within the product evens out over time.

### 3.1. Temperature Profiles: Average, Maximum, and Minimum Temperature Profile

Figure 3f presents the simulated volume average temperature (*Tavv*) of the salted pork loin during the drying–smoking, steam-cooking, water-cooling, and air-cooling operations. The temperature of the product increases during the first stage (drying–smoking) and then suddenly decreases for a short time. The short break period occurs during the transfer of the product from the smoking chamber to the steam chamber, where the product is exposed to ambient conditions. This means that the transfer time should be as short as possible to reduce energy loss and avoid a drop in the product temperature. The product temperature increases rapidly in the steam chamber until the temperature is closer to the steam temperature and then followed by the rapid cooling of the product until it is transferred to the cooling room. The product slowly cools down in the cooling room. The latter is due to the lower heat transfer coefficient between the air and the product compared to the higher heat transfer coefficient between the water and the product.

Figure 3f presents the hot spot (maximum temperature) and cold spot (minimum temperature) of the product. The figure illustrates the temperature range within the product at each time during all the processing steps. The cold spot is at the centre of the product during the heating processes (drying–smoking and steam-cooking), whereas it is at the surface of the product during the cooling processes (water-cooling and air-cooling), as illustrated by the modelling of the time–temperature distribution within the product in Figure 3. Similarly, the hot spot is found at the surface during the heating process (Figure 3a–c) and at the centre during the cooling process (Figure 3d). At the end of the water-cooling processes, the curve of the cold spot shows a constant temperature profile for a short period during the transfer time from the water-cooling process in the cooking chamber to the cool room. However, this is not observed from the average temperature profile curve. This clearly illustrates that the numerical simulation helps to track the cold spot and hot spot during all the process steps, which is not easy using the temperature sensors.

### 3.2. Model Validation and Effect of Position on the Temperature Profile

**Figure 4:**
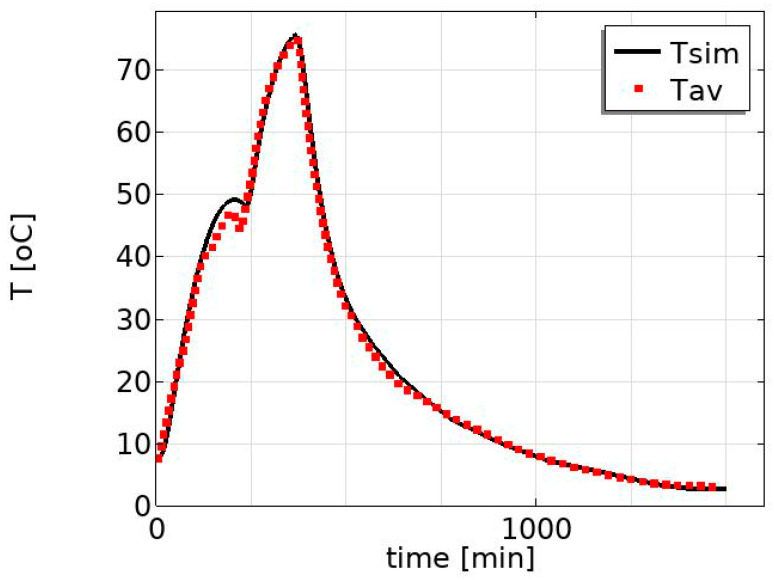
Validation of temperature profiles: the average measured temperature (the dotted line) and the simulated temperature profile (the solid line). The average measured temperature is the average of the 16 measured temperature profiles at the center of the product where the products are placed in different positions on pallets (see Figure 2).

The model was validated by comparing the simulated and measured temperature profiles at the center of the product. Figure 4 shows the measured average temperature profile and the simulated temperature profile. There is a good agreement between the average measured temperature (dotted line) and the simulated temperature profile (solid line). The average measured temperature is the average of the 16 measured temperature profiles at the center of the product where the products are placed at different positions on pallets, as illustrated in Figure 2.

**Figure 5:**
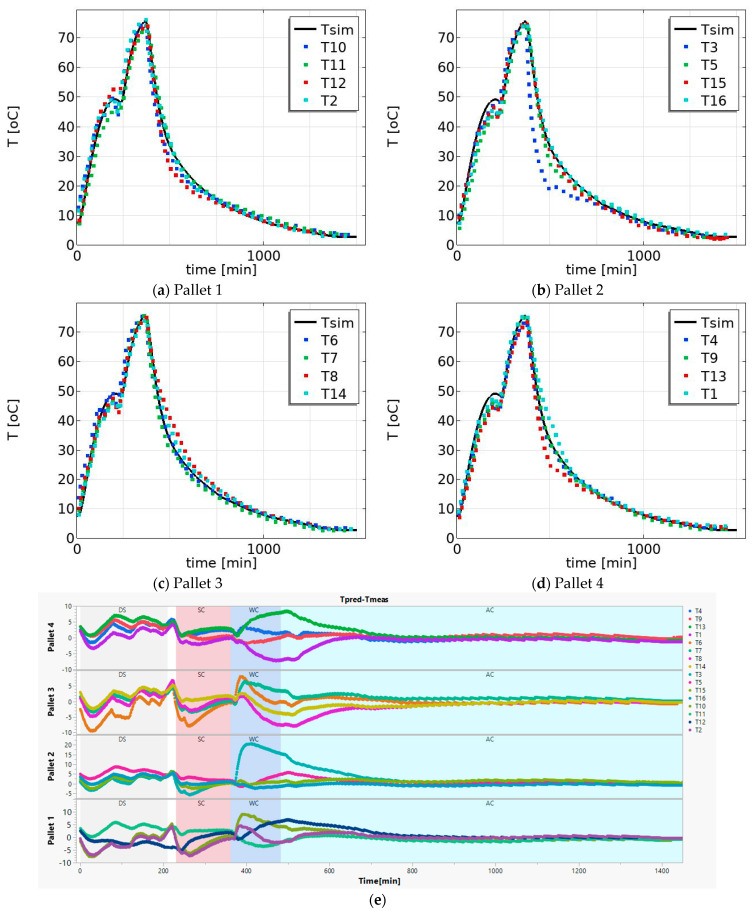
(**a**) Validation predicted temperature (solid line); measured temperature (dot lines) at: (**a**) pallet 1 (T12, T10, T11, and T2), (**b**) pallet 2 (T3, T5, T15, and T16), (**c**) pallet 3 (T7, T6, T14, and T8), (**d**) pallet 4 (T13, T4, T1 and T9). (**e**) The difference between model prediction (*Tpred*) and observation (*Tmeas*) as function of time for all the cases (pallets and locations), (see Figure 2 for the position). Only one of the temperature profiles (T11) was employed in the model to estimate the heat transfer coefficient (i.e., used for calibration to determine *h_air_* and *h_w_*). The remaining positions were utilized for validation purposes.

For each pallet, there are four measured temperature profiles at four different positions—from the top to the bottom, as shown in Figure 2. The measured data and the predicted temperature profile at center of the product were compared for different pallets. Figure 5 presents the measured and the simulated temperature profiles for pallets 1, 2, 3, and 4. A strong alignment is evident between the measured temperature (depicted by the dotted line) and the simulated temperature profile (illustrated by the solid line); see Table 2 and Figure 5e. Furthermore, this alignment provides support for the idea that the estimated parameters (*h_w_* and *h_air_*) obtained from one of the measurements, namely T11 maintain reasonable validity.

The product temperature profile varies among the pallets due to the location of the pallets. This is illustrated as follows: the majority of the measured temperature profiles are higher than the simulated temperature on pallet 1 (Figure 5a) in the drying–smoking region, whereas the majority of the measured temperature profiles are lower than the simulated temperature on pallet 2 (Figure 5b) in the drying–smoking region. This is due to variations in the airspeed and process temperature at different positions within the drying–smoking chamber and the airspeed is varying at different locations as a result of geometry for flow in the chamber. The variation in the temperature profile during the steam-cooking process is the smallest among the four regions. This is due to the higher heat transfer coefficient in the steam-cooking process. The model simulation also confirms that the variation in heat transfer coefficient during the steam cooking does not influence the centre product temperature profile significantly, as can be seen in Figure 5. In most cases, the predicted temperature profiles are higher than the measured values for the layers at the bottom, while they are lower for the top layer (as shown in Figure 5e).

**Figure 6:**
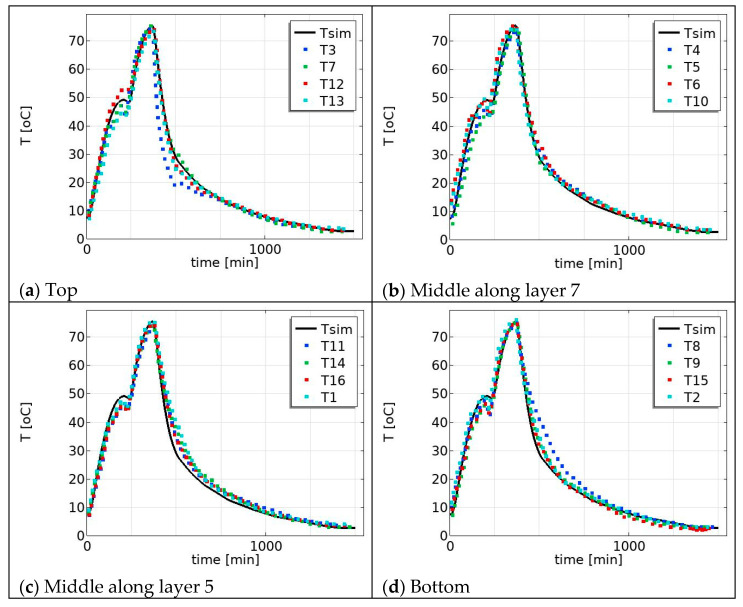
Predicted temperature (solid line) and measured temperature (dot lines): (**a**) top: along layer 10 (T12, T3, T7, and T13 in Figure 2a); (**b**) middle: along layer 7 (T10, T5, T6, and T4 in Figure 2a); (**c**) middle: along layer 5 (T11, T16, T14, and T1 in Figure 2a); and (**d**) bottom: along layer 3 (T2, T15, T8, and T9 in Figure 2a).

**Figure 7:**
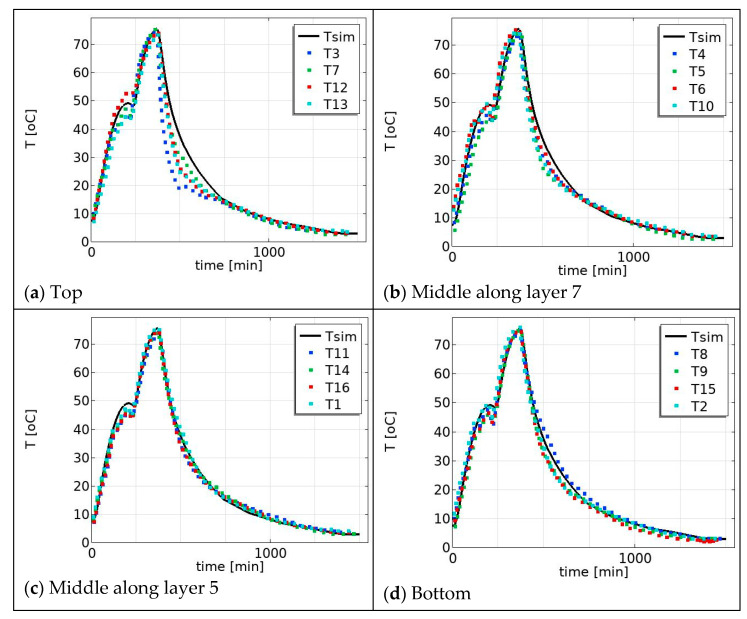
Predicted temperature (solid line) and measured temperature (dot lines): (**a**) top: along layer 10 (T12, T3, T7, and T13 in Figure 2): (**b**) middle: along layer 7 (T10, T5, T6, and T4 in Figure 2a); (**c**) middle: along layer 5 (T11, T16, T14, and T1 in Figure 2a); and (**d**) bottom: along layer 3 (T2,T15, T8, and T9 in Figure 2a).

The product temperature profile also varies with the position in the pallet, for example from the top to the bottom of the pallet, Figure 6. In the water-cooling region, the measured temperature profiles for the middle–top (i.e., along layer 7 in the Figure 2a) are in good agreement with the simulated temperature profile (Figure 6b), but the measured temperature profiles for the far top layer (layer 10) are slightly below the simulated temperature profile (Figure 6a) and the measured temperature profiles for the bottom layer are above the simulated temperature profile (Figure 6d). Particularly during the water-cooling process, the product in the top position cools faster compared to the product in the bottom position. This is due to the cooling water temperature at the top of the pallet being colder than at the bottom of the pallet as a consequence of the design of the cooling procedure. The cooling water temperature is heated up as it goes down along the pallet due to the heat release from the product. For this reason, we varied the process temperature (water temperature) in the simulation to mimic the measured temperature variation and to investigate the effect on the product temperature profiles. The process temperature (water temperature) was varied (25 ± 5 °C) and the effect on the simulated temperature profiles was compared in Figure 7. Figure 7a shows the measured temperature profiles for the top layer and the simulated temperature for the lower process temperature, Tw = 293 K, which is 5 °C lower than the temperature used in the previous simulation presented in Figure 6a. Figure 7d shows the measured temperature profiles for the bottom layer and the simulated temperature for a higher process temperature, Tw = 303 K, which is 5 °C higher than the simulated results in Figure 6d. In the water-cooling stage, there is better agreement between the simulated (with condition Tw = 25 ± 5 °C) and the measured data in Figure 7. This suggests that incorporating this temperature condition (i.e., reduction cooling medium as flow down) into temperature predictions during the cooling stage results in more accurate and realistic simulations, which justifies our assumption. Further, the effect can be more comprehensively modeled by integrating it with flow dynamics and accounting for all geometric configurations, including heterogeneity, using computational fluid dynamics (CFD). Such an approach has the potential to offer deeper insights; however, it may come at the cost of increased computational intensity, making it less suitable for industrial food manufacturing process control and monitoring.

Overall, the model obtained describing multistage processing can be used in the prediction of temperature profiles throughout processing operations (at all stages), for optimization, control, and monitoring of industrial meat processing, and it can further be integrated with the microbiological model or kinetics of microbial inactivation for the prediction of food safety.

## 4. Conclusions and Perspective

The mathematical model of heat transfer dynamics of multi-stage processing during operations during the processing of salted–smoked pork loin was obtained. The multistage process operations encompass different stages of processing: drying–smoking, steam-cooking, water-cooling, and air-cooling, and transferring stages. The developed model provides better insight on the heating and cooling of the salted–smoked pork loin by predicting the time–temperature profile or distribution. A good agreement between the measured industrial data and the predicted temperature profile was obtained at different positions in the heating, steam-cooking, and cooling chamber. This leads to the conclusion that the developed model of heat transfer is suitable for describing the heating and cooling processes of the production of salted–smoked pork loin. The obtained model can be used to predict the temperature profile–temperature as a function of position and time, particularly cold and hot spot temperature profiles for entire processes, and, thereby, can be a valuable tool in relation to food safety concerns. The model can also be used to study the effect of different parameters/setups on the product temperature profile, and thereby be a valuable tool for process optimization. From an industrial perspective, the development of a simulation tool capable of predicting temperature profiles at different stages of food processing is a significant step toward the digitization of food manufacturing. In this way, the utilization of such a model has the potential to not only improve food production processes, but also contribute to the development of sustainable and environmentally friendly practices in the food industry.

## Figures and Tables

**Table 1:** Model input parameters.

Parameter	Value [Unit]	Description	Source
D	0.085 [m]	Diameter of the product	This study (measured)
L	0.54 [m]	Length of the product	This study (measured)
*t_ds_*	12,600 [s]	Total time for the drying–smoking process (*t_d_* + *t_smoke_*)	This study (measured)
*h_d_*	20 [W/(m^2^K)]	Heat transfer coefficient during drying and smoking process	[21,22]
*T_d_*	333.15 [K]	The temperature of the air during the drying process	This study (measured)
*T_smoke_*	318.15 [K]	Process temperature during the smoking process	This study (measured)
*h_smoke_*	*h_d_*	Heat transfer coefficient during the smoking process	Assumed the same as during drying
*t_st_*	7920 [s]	Time for the steam-cooking process	This study (measured)
*h_st_*	2000 [W/(m^2^K)]	Heat transfer coefficient during the steam-cooking process	[23]
*T_st_*	351.15 [K]	The steam temperature during the steam-cooking	This study (set+)
*t_w_*	7200 [s]	Time for the water-cooling process	This study (measured)
*h_w_*	80 [W/(m^2^K)]	Heat transfer coefficient for the water-cooling process	This study (estimated using measured data, Section 2.3))
*T_w_*	298.15 [K]	Water temperature during the water-cooling process	This study (measured)
*t_air_*	54,000 [s]	Time for the air-cooling process	This study (measured)
*h_air_*	12 [W/(m^2^K)]	Heat transfer coefficient during the air-cooling process	This study (estimated using measured data, Section 2.3)
*T_air_*	276.15 [K]	Air temperature during the air-cooling process	This study (measured)
*T_o_*	280.65 [K]	The initial temperature of the product	This study (measured)
*t_trans1_*	1200 [s]	Transfer time (transferring the product from DS to SC)	This study (measured)
*t_trans2_*	1200 [s]	Transfer time (transferring the product from SC to AC)	This study (measured)
*T_amb_*	298.15 [K]	The ambient air temperature during the transfer time	This study (measured)
*h_amb_*	8 [W/(m^2^K)]	Heat transfer coefficient during the transfer time	[14]
*ρ_m_*	ρm=1∑xiρi ** 1064.5 [kg/m^3^]	The density of the product	Estimated from composition [3,16,24]
*cp_m_*	$c_{p,m}=\sum c_{p,i}x_{i}$, ** 3535.5 [J/(K kg)],	Specific heat capacity of the product	Estimated from composition [3,16,24]
*k_m_*	km=kmix=gkper+1−gkpar where kparkpar=∑εi kikper=1∑εiki ** 0.47 [W/(m K)]	Thermal conductivity of the product	Estimated from composition [16,25]

*ρ_i_* is the density (in kg/m^3^) of each component *i* (water, fat, carbohydrate ash, and protein), *x_i_* is the mass fraction of each component *i*, *ε_i_* is the volume fraction of each component *i*, *k_per_* is the thermal conductivity based on the perpendicular model, and *k_par_* is the thermal conductivity based on the parallel model. ** The indicate the typical values calculated using the equation for this. The model calculates based on the composition using equations shown in Table 1.

**Table 2:** R^2^ and RMSE of model prediction against measured data (at different positions 1–16, see Figure 2). The one with * is used for the parameters estimation.

	*T3*	*T4*	*T5*	*T6*	*T7*	*T8*	*T9*	*T10*	T11 *
***R^2^***	0.938	0.997	0.989	0.985	0.994	0.987	0.996	0.982	0.992
***RMSE***	5.945	1.697	2.895	2.653	2.013	2.672	1.639	2.881	2.047
	***T12***	***T13***	***T14***	***T15***	***T16***	***T1***	***T2***	***Tav***	
***R^2^***	0.987	0.989	0.994	0.996	0.995	0.991	0.991	0.997	
***RMSE***	2.495	3.244	1.751	1.773	1.590	2.263	2.152	1.260	

## Data Availability

The data presented in the study may be available on reasonable request from the corresponding author.
